# A systematic review of the evidence of outdoor air pollution on asthma hospital visits in children and adolescents in South Asia – a call for data

**DOI:** 10.12688/wellcomeopenres.16991.1

**Published:** 2021-07-06

**Authors:** Sowmya Malamardi, Katrina A. Lambert, Mehak Batra, Rachel Tham, Mahesh Padukudru Anand, Bircan Erbas

**Affiliations:** 1 School of Psychology & Public Health, College of Science Health and Engineering, La Trobe University, Melbourne, 3086, Australia; 2 Department of Respiratory Medicine, JSS Academy of Higher Education & Research (JSSAHER), Mysuru, Karnataka, 570023, India; 3 Mary MacKillop Institute for Health Research, Australian Catholic University, Melbourne, 3086, Australia; 4 Faculty of Public Health, Universitas Airlangga, Surabaya, Indonesia

**Keywords:** asthma, air pollution, South Asia, hospitalization

## Abstract

**Background:** Outdoor air pollution and childhood asthma are increasing problems in South Asian countries. However, little is known about the associations between levels of air pollution and severe childhood asthma requiring hospital treatment in these regions.

**Methods:** We undertook a systematic review to assess the evidence between outdoor air pollution exposure and childhood and adolescent asthma hospitalization in South Asia. MEDLINE, Web of Science, Google Scholar, CINAHL, Embase, Scopus, ProQuest Central databases were searched for peer-reviewed papers, and examination of reference lists was conducted for additional studies. We identified all the literature published in English up to January 2021 for the study population comprised of children aged less than 19 years. The search strategy was designed to identify all the studies and screen them as per the inclusion criteria. The method of qualitative synthesis using the standard tool determined the comprehensiveness of the assessment of bias.

**Results:** Of the original 367 studies screened three studies were ultimately included from India, Pakistan and Sri Lanka and a narrative synthesis was conducted. Although studies reported adverse effects of outdoor pollution on asthma hospitalizations, limitations in exposure assessments, varying definitions of asthma hospitalizations and limited data analysis were identified.

**Conclusions:** There is currently limited evidence that can provide meaningful risk estimates of the impact of outdoor air pollution on asthma hospitalizations during childhood and adolescence. Studies with comparable outcome definitions, appropriate exposure assessments and study designs are needed to inform future public and environmental health policy.

**PROSPERO registration:** CRD42020156714 (28/04/2020)

## Introduction

Asthma is an important global health issue affecting people of all age groups and is one of the most common non-communicable diseases (NCD). Asthma prevalence is increasing and globally, it is the 16 ^th^ leading cause of years lived with disability (YLD) among all ages and the 4 ^th^ leading cause among children aged 5–14 years ^1^. Children bear the highest direct costs associated with asthma (hospital visits, doctor visits, diagnostic tests, and medication) as well as indirect costs such as school and work days lost due to illness and care responsibilities and impaired quality of life, or sometimes, premature death ^2^. With this growing trend, it is estimated that by 2025, there will be one hundred million additional cases of asthma ^3^. Although respiratory diseases are expected to be the second highest contributor to economic burden by 2025, developing countries are frequently short of reliable estimates due to limited health system diagnosis and reporting, and impaired quality of care ^4, 5^. Afghanistan, Bangladesh, Bhutan, Maldives, Nepal, India, Pakistan and Sri Lanka are developing countries in South Asia where the prevalence of asthma is increasing, but very few have been studied to understand the strongest risk factors leading to this situation ^6, 7^.

The overall prevalence of asthma in Asia is 3.5% ^3, 4^ and has increased ^8^ due to growing population, increased prevalence of chronic NCDs, increasing urbanization and rising air pollution levels ^4, 8– 10^. In South Asia, the overall prevalence of asthma among children aged < 19 years is 1.74% ^11^. However there are wide variations across countries, for example, 1.59% for Bangladesh and 6.14% for Afghanistan ^11^. Studies in Canada and the United Kingdom (UK) have observed an increase in hospitalization among children and young adults of South Asian ethnic groups over a period of 9 years ^12– 14^.

Changes in environmental conditions including increasing urbanization, energy use and vehicular traffic are increasing and may contribute to asthma hospitalizations ^15, 16^. Lifestyle factors such as smoking and obesity, along with environmental exposures which can include aeroallergens (pollen, fungal spores, dust, animal dander), air pollutants and respiratory viruses, individual characteristics such as being male and having genetic predispositions are associated with asthma hospitalizations in children and adolescents ^17– 19^. In terms of major environmental factors, short and long-term exposures to air pollutants such as coarse particulate matter (PM) sized <10μm in aerodynamic diameter (PM _10_), fine PM sized <2.5μm (PM _2.5_), ultrafine PM (UFP), ozone (O _3_), volatile organic compounds, hydrocarbons, nitrogen dioxide (NO _2_) and sulfur dioxide (SO _2_) may act as triggers for asthma exacerbation which may lead to hospitalization ^20– 22^. South Asian countries are predominantly agrarian countries with extensive crop burning; have increasing motor vehicle use and rapid industrial development contributing to high levels of air pollution. The permissible levels of air pollutants set by public authorities varies across South Asian countries and cities ^23^ and the evidence around health effects remains inconclusive across multiple geographical locations and sources of emissions. Children residing in the developing countries of South Asia have a greater risk of severity of disease and mortality due to asthma due to their developing lungs, and high volume of air intake relative to their size and their preponderance to engage in outdoor physical activities ^24– 26^. Asthma exacerbations in children are associated with poor lung growth ^27^ which in turn is associated with reduced longevity over their life course ^28, 29^. Hence children’s respiratory health is critical for their long-term health and wellbeing but little is known about the impact of outdoor air pollution in these geographic locations.

To address this knowledge gap, we conducted a systematic review to assess the current evidence on the relationship between outdoor air pollution and asthma hospitalization of South Asian children and adolescents.

## Methodology

This review is registered with the International Prospective Register of Systematic Reviews (PROSPERO Registration no. CRD42020156714, 28 ^th^ April 2020) and we followed the Preferred Reporting Items for Systematic Reviews and Meta-Analyses (PRISMA) guidelines for conducting reviews ^30^. The inclusion criteria were analytical research articles published in English up to January 2021 with the study population comprising children aged less than 19 years. In studies with adults and children, data specific for children needed to be presented separately in order to be included in the review; studies were conducted in a South Asian country which includes Afghanistan, Bangladesh, Bhutan, Maldives, Nepal, India, Pakistan and Sri Lanka; and studies must have explored at least one outdoor or ambient air pollutant and its relationship to asthma exacerbations requiring attendance to a hospital. There were no restrictions on the study design.

A comprehensive literature search was conducted using the electronic databases: MEDLINE, Web of Science, Google Scholar (first 10 pages), CINAHL, Embase, Scopus and ProQuest Central, using combination of key terms and MeSH terms (Extended data, Table 1 ^30^). Additional studies were included by examining the references of screened studies. After removing the duplicates, screening titles and abstracts, papers were identified for full text review and their eligibility was independently tested by two authors (SM and MB). Any conflicts between the two reviewers were then discussed with other coauthors before extracting the data.

Details of type of study design, location of study, study settings, year the study was conducted, age range and number of children studied, data on the exposure (primary and secondary considered), outcome, risk estimates with 95% confidence intervals (95% CI), confounders and adjustment covariates, or effect modification or interaction were extracted from the studies.

A quality assessment was conducted on the eligible studies using a validated quality assessment tool by Zaza *et al*., 2020 with that evidence the quality of study based on study design, study sample, exposure assessment, outcome measures and adjustment for confounders ^31^. Two authors (SM and MB) independently assessed each article, scored, and any differences resolved by consensus and inputs by other co-authors. A benchmark score of 75% and above as high-quality studies and below 75% set as low-quality studies.

## Results

**Figure 1.**
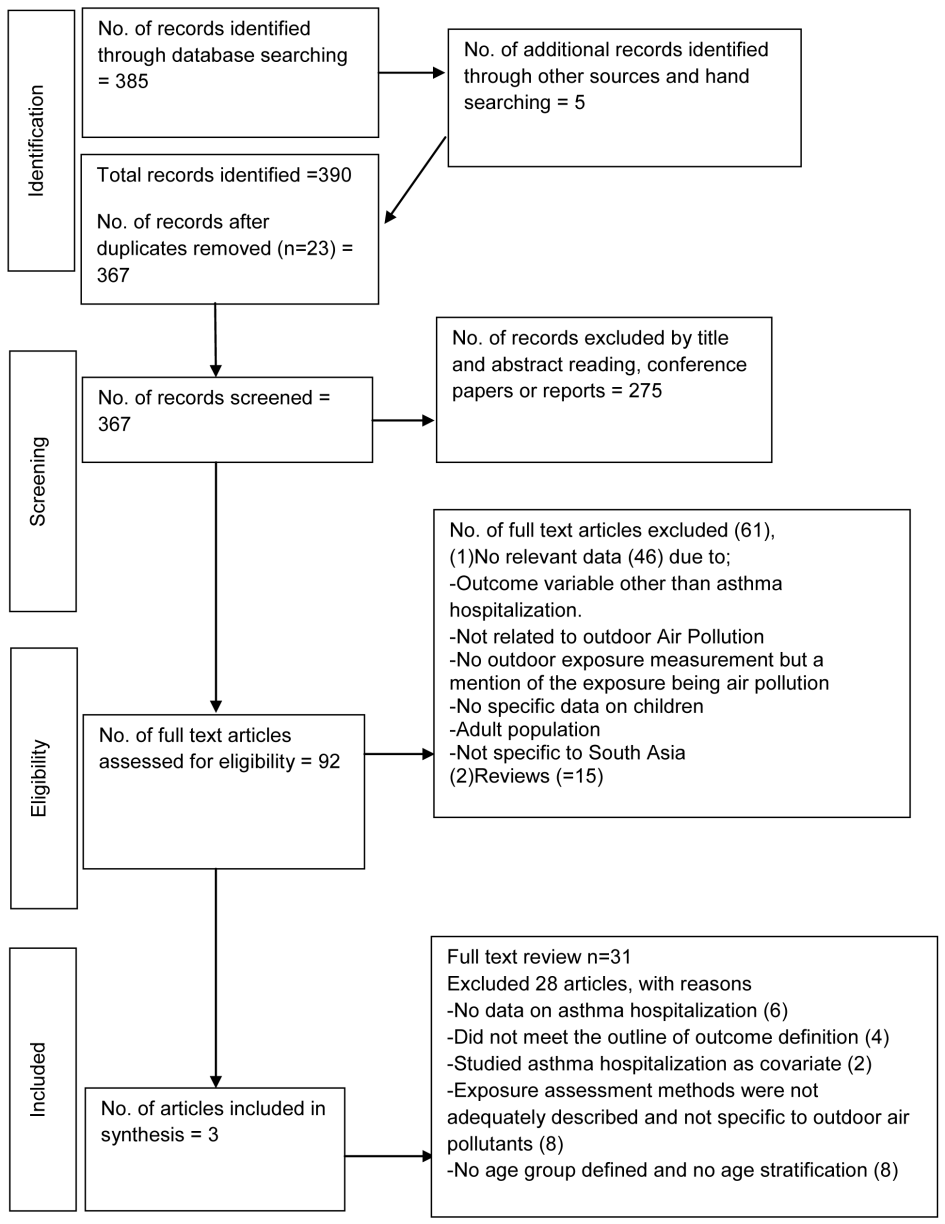
Preferred Reporting Items for Systematic reviews and Meta-Analyses (PRISMA) flow diagram of the included studies in the review, January 2021.

The electronic search and manual search found 367 peer-reviewed scientific articles after removing the duplicates ( Figure 1). Of these, 275 were excluded following review of titles and abstracts. On conducting a full text review of the remaining 92 articles, all but three were excluded as they were found irrelevant and not meeting the inclusion criteria (Extended data, Table 2 ^30^). The primary reason for the exclusion of studies was not meeting the outcome definition. For example, Awasthi *et al*. ^32^ reports 157 of the hospitalized 386 cases were for asthma, but they treat this variable as a covariate, not an outcome. Similarly, in Singh *et al*. ^33^ while the asthmatic cohort after hospital admission enrolled in the study, the prospective six-month study outcome related to air pollution exposure was “asthmatic episode” or “asthma exacerbations” that not necessarily relates to another hospitalization. Also, the authors using the terms interchangeably and do not define them clearly ^33^. Studies in other countries such as Bangladesh reported asthma prevalence and air pollution associated asthma diagnosed by a physician but did not include asthma hospitalization ^34^. The secondary exclusion was the lack of outdoor air pollution exposure assessment. Some studies were excluded because they did not state the age group of the study participants ^35^ or did not report age stratified analyses specific to children ^36^. No relevant studies met the inclusion criteria from Afghanistan, Bangladesh, Bhutan, Maldives, Myanmar, Nepal or Sri Lanka. Only three papers were ultimately included as they fulfilled all inclusion criteria. Only one study scored fairly well in the quality assessment with a difference in the methodology as a measure of exposure, appropriate statistical methods used, controlling for key confounding variables and interpretation of findings. We were unable to conduct a meta-analysis of the results; instead we conducted a narrative synthesis of the results and present key issues pertaining to the other studies that met the initial search criteria at full text review.

The characteristics of the included studies are presented in Table 1. The three studies of South Asia were conducted in child populations from Kolkata *,* India ^37^, Lahore, Pakistan ^38^ and Colombo, Sri Lanka ^39^. In the Kolkata study children aged 5 to 18 years and in Lahore study only 2 to 15-year-old were included whereas in Colombo study all children less than 12 years were part of the study. All had a substantially large sample size and used time series analyses to assess the association between outdoor air pollution and the asthma hospitalization. Chakraborty *et al*. ^37^ included both Internal Classification of Diseases 10 (ICD-10) codes J45, J46, and ICD-9-CM code 493.90. The frequency of hospital admissions following an asthma attack or exacerbations were reported in Anwar *et al*. ^38^ Senanayaki *et al*. ^39^ reported the total number of visits to an emergency treatment unit (ETU) made by children presenting with acute wheezing episodes.

**Table 1.** Characteristics of the included studies from South Asia evaluating outdoor air pollution concentrations and hospital admission due to asthma in children. ICD=Internal Classification of Diseases.

Author, study design and analysis methods	Location of study (City, District, and Country	Study Population	No. of children in the sample	Age group	Exposure (Pollutants) measurement	Exposure measurement	Outcome definition
Chakraborty *et al*., (2014) Time series; Generalized Additive models (GAM).	Kolkata, West Bengal, India	Children attending the emergency department of Sir Nilratan Sarkar Hospital and National Medical College Hospital due to asthma exacerbation, between 2009–2010	2,708	5–18 years	Particulate matter sized <10micro meter (PM10) (μg/ m ^3^) and Ozone (O _3_) (ppb)	Daily concentrates (PM _10_) and Ozone (O _3_) collected from West Bengal state pollution control board, India. Location: Victoria Memorial, Kolkata (approx. 10 km from hospitals.)	Asthma hospitalization: ICD-9-CM code 493.90; ICD-10 code J45/4d first discharge diagnosis: Emergency hospital admission due to asthma during 2008–2010
Anwar *et al*., (2012); Time series; Frequency distribution and measure	Lahore, Pakistan	Children visiting the physician at Mayo Hospital and Children hospital at Lahore, Pakistan following an acute asthma exacerbation.	1135	2–15 years	Total soluble particles (TSP) µg/m ^3^, Nitrogen dioxide (NO _2_), Nitric Oxide (NO), Sulfur dioxide (SO _2_) in ppb and carbon monoxide (CO) ppm.	Monthly data on pollutants TSP, NO _2_, NO, SO _2_, and CO were collected from Emergency Protection Agency department during 2002 to 2003. Location: Urban Lahore, Pakistan	Hospital visits due to asthma and in acute asthma exacerbation cases
Senanayake *et al*., (2001); Case control study; Correlational Analysis	Colombo, Sri Lanka	Children presenting with acute wheezing and no wheezing episode attend the emergency treatment unit (ETU) of Lady Ridgeway Hospital (LRH). Visits made between 1 ^st^ July 1998 to 30 ^th^ June 1999 by the children attending ETU with or without wheezing were studied.	Total 41,032 of children of which Cases=30,932 (with wheezing) Controls=10100 (without wheezing)	< 12 years	Sulfur dioxide (SO _2_) (ppm) and oxides of nitrogen (NO _x_) in ppm	Daily hourly concentrates of pollutants SO _2_ and NO _x_ collected from air quality monitoring station of Environmental Division of the National Building Research Organization (NBRO), Central Environmental Authority of Sri Lanka. Location: Colombo Fort, Sri Lanka (approx. 3 km from Lady Ridgeway Hospital)	Emergency hospital visits made following an acute episode of wheezing associated asthma for emergency care and management

The outdoor air pollutants measured were NO _2_ (in parts per billion- ppb) _,_ SO _2_ (ppb), O _3_ (ppb), CO (ppb), PM _10_ (µg/m ^3^), and total suspended particles (TSP) (µg/m ^3^). The three studies reported indirect measure of population exposure to air pollutants measured at fixed monitoring stations as determined by the respective national pollution control board depending on the region. The findings of these studies are shown in Table 2. Chakraborty *et al*. ^37^ studied associations between with daily concentration levels of PM _10_ and O _3_ obtained from the monitoring station of the West Bengal pollution control board over 12 month period and asthma hospitalizations of children aged 5–18 years ^37^. This daily time series study used generalized additive models (GAM) to assess non-linear relationship among pollutants PM _10_ and O _3._ They found that PM _10_ is associated with increased emergency asthma hospitalization in school aged children [0.0037, 0.007 (estimate, SE), p<0.01]. They also reported associations with O _3_ [0.0022, 0.001 (estimate, SE), p<0.01]. They did not report the estimated effects as they modelled the non-linear smooth associations only. The authors adjusted for confounding factors such as humidity, temperature and wind speed. The study did not report any interactions between confounding variables for childhood asthma hospitalization ( Table 2).

**Table 2.** Findings from the included studies from South Asia evaluating outdoor air pollution concentrations and hospital admission due to asthma in children.

Author	Findings/ Risk estimates	Covariates included in the models as Confounders	Interactions
Chakraborty *et al*. (2014)	Generalized additive models were used to analyze the daily time series data. No direct estimates were provided as they were smoothed. Estimate of particulate matter sized <10micro meter (PM _10_): LOESS method-0.0010 (0.0008), p<0.01 and by Spline method is 0.0037 (0.0007), p<0.01 Estimate of Ozone (O _3_): LOESS: 0.0024 (0.0018), p= 0.82 Spline method is 0.0022 (0.0011), p=0.0024. In addition, significant seasonal variation in daily asthma hospitalization was observed. There were two peak seasons, the first one (up to 24/day) was in spring- early summer (last week of March to mid-April) and the second one (up to 23/day) was in autumn (mid-September to October)	Temperature, Humidity, Rainfall and season.	None
Anwar *et al.* (2012)	No statistical modelling conducted. The recorded cases were proportionately high consistent with the higher concentration of SO _2_ and total soluble particulates (TSP) in the month of July to December and January respectively.	Although the weather data were not considered as confounders asthma attendance were high in the months of July to December corresponding to high humidity and lower temperature.	None
Senanayake *et al*., (2001)	Estimated concentration of pollutants varied but followed a consistent pattern, Correlation co-efficient (r>0.6) and the estimates are SO _2_: 0.1- 0.01 parts per million (ppm) NO _2_: 0.28- 0.04 ppm Highest visits to the emergency treatment unit (ETU) were made on maximum pollutants (both SO _2_ & NO _2_) concentrations days. (p= 0.05) which co-occurred 16 times in 41 weeks study of SO _2_ and 13 times in 47 weeks study of NO _2_ among cases. The lowest no of visits per day to emergency treatment unit (ETU) associated with least daily pollutant concentrations. Such a presentation manifested 22 events in 48 weeks and 27 events in 49 weeks across SO _2_ and NO _2_ among cases.	None	None

Anwar and colleagues ^38^ estimated the measure of pollutants: TSP, NO _2_, NO, SO _2_, and CO using data from the Environmental Protection Department, Lahore, Pakistan. In a study sample of 1135 children aged 2 to 15 years during October 2002 an increase in SO _2_ concentration of 80 ppb was associated with increased number of asthma emergency visits. No statistical modelling was used to analyze the associations. They did not provide any age or sex stratification when describing the study participants ^38^.

In Colombo, Senanayake *et al*. ^39^ examined the children attending the ETU for asthma management (N=30,932) and for other than asthma (N=10,100). In the study, estimated concentration levels of SO _2_ and oxides of nitrogen (NO _x_) coincided with the frequency of the hospital visits and the effect estimates for SO _2_ and NO _2_ were 0.1- 0.01 parts per million (ppm) and 0.28- 0.04 ppm respectively. The weekly pattern of hospital emergency visits for asthma correlated with days of high pollutant concentration. This study reported correlation results only and did not perform any statistical modelling nor did they adjust for seasonality, age group and gender.

We conducted a quality assessment of the three studies and found significant issues with quality of reporting and risk of bias inherent in the studies. Overall, the outcome variable definition was unclear, and they conducted limited statistical analyses ^38^. Single monitoring station data were used in each study contributing to risk of exposure assessment misclassification ^37– 40^. In known cases of asthmatic children, it is not unusual to have repeated asthma attacks leading to hospitalization; these studies did not discuss the issue of repeat admissions. Chakraborty *et al*. ^37^ reported the seasonal variations in the regression models but did not adjust for seasonality, gender or age.

## Discussion

Initially, we sought to conduct a systematic review but after implementing the search, screening and conducting the eligibility check only three studies were eligible for inclusion. To our knowledge, this is the first review that has attempted to comprehensively synthesize the evidence, assessed the strengths, limitations and quality of studies reported from South Asia on the effect of outdoor air pollution and asthma related hospitalizations in children and adolescents. Although the three studies indicated some adverse effects, this review identified significant deficiencies in exposure assessments and outcome definitions.

The ecological time series study conducted in Kolkata by Chakraborty *et al*. ^37^ was one of the few studies with clear definitions of outcome definitions and 5 to 18 years age group and assessment of air pollutants from monitoring stations ^37^. They had sufficient sample size (n=2,708) to conduct a rigorous statistical analysis which captured the nonlinear relationships between PM _10_ exposure and the outcome. Reasonable adjustments for confounding effects of temperature were also considered but no interaction or age, sex strata analyses were presented. The study conducted in Lahore was a cross sectional descriptive study, but we classified it as an ecological study due to the trend of hospitalization related to asthma were studied ^38^. On the other hand, the Colombo study was classified as time series though the author reported it as a case-control study ^39^. These three were focused on children and adolescents residing in urban areas. Although there were other studies in the initial search with exposure measures from monitoring stations such as Singh *et al*. ^33^ the outcome definitions were not related to a hospital visit or admission because of asthma.

Seasonality is an important variable to consider when assessing outdoor air pollution exposure and asthma hospitalizations. Studying the effect of periods involving different seasons and weather conditions was not an objective of the included studies ^38, 39^. The findings from two of the three included studies show that particulates PM _10_ and TSP were high in the month of summer ^37, 38^. Although most of the asthma-related hospitalization occurred in winter, they also peaked in summer in Chakraborty *et al*. ^37^ and during the transition from one season to another corresponding to October and November as an autumn season in a study by Anwar *et al*. ^38^. Seasonal variation is influenced by other environmental factors such as temperature, humidity, rainfall, wind, pollen grains, fungal spores, viral infections and others. The study at Kolkata controlled for the meteorological factors; average temperature, humidity and wind speed in the model ^37^. Spore concentration per day was adjusted along with covariates PM _10_ and O _3_ in the model and thus spore counts (per cubic meter per day) were associated with increased asthma hospitalizations during spring and autumn ^37^. The effect of temperature was important in both studies but only Chakraborty *et al*. ^37^ adequately modelled it. Neither study adjusted for other variables such as exposure to environmental tobacco smoke (ETS), dust allergens, household ventilation, history and current smoking. Moreover, they did not assess gender and age-specific effects.

Our review included a comprehensive systematic search of eight electronic databases, additional hand searching, and other narrative literature reviews on similar topics in these regions, hence presents unique results but with limited evidence *.* Although the search terms did include all countries in South Asia, most of the studies that fulfilled the initial search criteria were from India. Studies from Sri Lanka were excluded as their defined study population age varied widely, ranging from infants to adults ^40^. Though, respiratory admissions including asthma were described in studies from Nepal but no results or discussion specific to asthma were presented ^41, 42^. Studies in our search from Pakistan and Bangladesh, described hospital admissions due to asthma, but studying air pollutants as exposure of interest was not their primary objective ^35, 43^. Developing countries facing economic constraints with the growing burden of asthma morbidity may experience greater challenges in the coming years ^44^. It is worth understanding that there is a lack of studies in South Asian countries; therefore, there should be urgency among public health authorities in health impact assessment along with appropriate air quality measurement or modelling ^16^. There is a need to measure outdoor air pollutants on a real-time monitoring basis and studying trends of hospital admissions to fulfil current gaps; lack of long-term studies is also the highlight of our findings ^13^. Our review was limited to studies in English language which might have missed some eligible studies published in other languages. But this is unlikely given that majority of the research work in South Asian countries are published within English language journals *.* We have presented key limitations in assessing the findings of studies included in our review. Diverse methodologies, including some with no statistical analysis at all and different statistical methods to assess exposure and outcome associations in the studies, made it impossible to conduct a meta-analysis.

In summary, we have shown that the evidence on the impact of air pollution on asthma hospital visits in children and adolescence in South Asia is scarce and a call for data is a priority. Outdoor air pollution is increasingly problematic but because of inconsistencies in exposure assessments, asthma diagnosis and hospitalization coding, controlling for key confounders and statistical analytical methods we were unable to synthesize the current studies and provide clear evidence of such associations. Respiratory health effects from outdoor pollution will continue to be a growing burden and more rigorous studies in South Asia are urgently needed.

## Data availability

### Underlying data

All data underlying the results are available as part of the article and no additional source data are required.

### Extended data

Open Science Framework: Air Pollution and Asthma Hospital visits in Children_South Asia. https://doi.org/10.17605/OSF.IO/XQRCH ^30^.

This project contains the following extended data:

-   Online Resource 1.docx ( Table 1: Search Strategy)
-   Online Resource 2.docx ( Table 2: Studies from search history excluded from final synthesis with reasons)

### Reporting guidelines

Open Science Framework: PRISMA checklist for ‘A systematic review of the evidence of outdoor air pollution on asthma hospital visits in children and adolescents in South Asia - a call for data’. https://doi.org/10.17605/OSF.IO/XQRCH ^30^.

Data are available under the terms of the Creative Commons Attribution 4.0 International license (CC-BY 4.0).
