# Supplementary material for: A systematic review of the evidence of outdoor air pollution on asthma hospital visits in children and adolescents in South Asia – a call for data
Source: Wellcome Open Res. 2021 Jul 6;6:174. [Version 1] doi: 10.12688/wellcomeopenres.16991.1 (PMC8749950; doi:10.12688/wellcomeopenres.16991.1)
Supplement: Peer review history

# Peer review: Reviewer response for version 1

**DOI:** 10.21956/wellcomeopenres.18761.r47446

**Published:** 2022-01-07

**Authors:** Balamugesh Thangakunam

**Affiliations:** 1 Department of Pulmonary Medicine, Christian Medical College, Vellore, Tamil Nadu, India

Asthma and respiratory infections are being increasingly recognized as causes of childhood morbidity. One of the proposed reasons is increasing air pollution. The authors have attempted to find out whether there is sufficient medical evidence to correlate air pollution to asthma episodes in children in South Asia. Authors have done a reasonably well strategized method of evidence synthesis and came up with only three studies from the sub-continent.

 They found that all three studies had significant issues with the quality of reporting and risk of bias and had also used data from a single monitoring station. This review highlights the lacunae of data on the impact of outdoor air pollution on asthma hospitalizations during childhood and adolescence and the urgent need for well conducted studies.

Are the rationale for, and objectives of, the Systematic Review clearly stated?

Yes

Is the statistical analysis and its interpretation appropriate?

Yes

Are sufficient details of the methods and analysis provided to allow replication by others?

Yes

Are the conclusions drawn adequately supported by the results presented in the review?

Yes

Reviewer Expertise:

Tuberculosis, lung cancer

I confirm that I have read this submission and believe that I have an appropriate level of expertise to confirm that it is of an acceptable scientific standard.

# Peer review: Reviewer response for version 1

**DOI:** 10.21956/wellcomeopenres.18761.r44860

**Published:** 2021-08-25

**Authors:** Sanjeev Nair

**Affiliations:** 1 Department of Respiratory Medicine, Government Medical College, Thiruvananthapuram, Kerala, India

A systematic review in an important area, which is of growing importance due to the increasing morbididty and mortality due to air pollution in South Asia. The search has been done systematically, however the authors have stated the issue of scarcity of studies in this area and lack of standardisation in these studies. Most of the studies seeked to measure exposure in terms of environmental measurements from toutine measurement of air pollutions from centres close to the institution, which may not be the ideal measure of exposure at an individual level, and the association with outcome in Asthamtic children and adolescents. The association could have been better established if there was direct measurement of household level and individual level exposure to the children to air pollution and then association could be looked for with exacerbations. However, there is lack of data / published studies with this kind of data. With the limitations of available data, the authors have done what was possible in a systematic review. Studies have looked at individual measurements of air pollution in three of countries included in this study and the authors could refer to those studies to point out the opportunity for individual measurements of air pollution and establishing association with outcomes related to Asthma in future studies. ^1^

Are the rationale for, and objectives of, the Systematic Review clearly stated?

Yes

Is the statistical analysis and its interpretation appropriate?

Not applicable

Are sufficient details of the methods and analysis provided to allow replication by others?

Yes

Are the conclusions drawn adequately supported by the results presented in the review?

Yes

Reviewer Expertise:

Tuberculosis, COPD and Asthma, Air pollution

I confirm that I have read this submission and believe that I have an appropriate level of expertise to confirm that it is of an acceptable scientific standard.

## References

[1] : Household and personal air pollution exposure measurements from 120 communities in eight countries: results from the PURE-AIR study. *Lancet Planet Health* .4(10) : 10.1016/S2542-5196(20)30197-2 e451-e462 10.1016/S2542-5196(20)30197-2 33038319PMC7591267
